# Carbon and nitrogen stable isotopic data of premodern human skeletons from mainland Japan and the Ryukyu islands

**DOI:** 10.1016/j.dib.2021.107359

**Published:** 2021-09-16

**Authors:** Takumi Tsutaya, Minoru Yoneda

**Affiliations:** a Department of Evolutionary Studies of Biosystems, The Graduate University for Advanced Studies, Shonan Village, Hayama, Kanagawa, 240-0193, Japan; b The University Museum, The University of Tokyo, Hongo 7-3-1, Bunkyo, Tokyo, 113-0033, Japan

**Keywords:** Bioarchaeology, Human skeletons, Japan, Palaeodiet, Premodern period, Ryukyu islands, Stable isotope analysis, Premodern period, Ryukyu Islands

## Abstract

This dataset consists of carbon and nitrogen stable isotope ratios of bulk collagen extracted from 229 human skeletons from premodern Japan. All samples were derived from different individuals excavated from mainland Japan and the Ryukyu Islands. Most of the skeletal individuals were identified, sexed, and aged by physical anthropologists. Collagen samples were extracted from bone or root portion of tooth dentin. Collagen samples were measured by an elemental analyzer coupled to stable isotope ratio mass spectrometry. Stable isotope ratios are the useful proxy of palaeodiet, and this dataset can be used for dietary reconstruction of premodern people living in the Japan archipelago.

## Specifications Table

**Table:** 

Subject	Archaeology
Specific subject area	Stable isotope analysis Bioarchaeology
Type of data	Table
How data were acquired	Thermo Flash 2000 elemental analyzer, Finnigan ConFlo III interface, Thermo Delta V mass spectrometer, Calro Erba NA1500 elemental analyzer, Finnigan MAT ConFlo II interface, and Finnigan MAT 252 mass spectrometer
Data format	Raw
Parameters for data collection	Bone or dentin samples of archaeological human (*Homo sapiens*) skeletal remains from mainland Japan or the Ryukyu Islands were used. These individuals were buried during the premodern period. Most samples were derived from different adult individuals.
Description of data collection	Collagen was extracted from bone or dentin samples. Extraction protocols followed standard methodologies [1]. Carbon and nitrogen stable isotope ratios of the bulk extracted collagen samples were measured by using an elemental analyzer coupled to stable isotope ratio mass spectrometry (EA-IRMS).
Data source location	**Samples** Archaeological site name: Kumanashi(8), Uwano, Araya, Takasu-bouzawa, Satohama, Dainichikita, Hachiman-hayashi, Uozukou, Kamiizawa-o'ne, Hodokubo, Shiokawa, Anrakuji-Higashi, Komekuyama B, and Kyomachi from mainland Japan. Yacchino-gama, Isonoirime-gohairyobaka, Mekarukobo, Nakandakariyama, Ayafune, Nusuku, Suubaru, and Tonoshiro from the Ryukyu Islands. Details: Please see Table 1. **Institutions** Institution 1: University Museum, the University of Tokyo City/Town/Region: Bunkyo, Tokyo Country: Japan Institution 2: Environmental Chemistry Division, National Institute for Environmental Studies City/Town/Region: Tsukuba, Ibaragi Country: Japan
Data accessibility	Repository name: IsoArcH [2] Data identification number: https://doi.org/10.48530/isoarch.2021.006 Direct URL to data: https://doi.isoarch.eu/doi/2021.006
Related research article	M. Yoneda, I. Tayasu, R. Ishimaru, F. Hyodo, S. Kusaka, T. Gakuhari, T. Yumoto, Doitai kara mita Nihon-retto no shoku seitai no hensen (Stable isotopic evidence of chronological changes in diet of ancient Japan). In: M. Takahara, N. Murakami, editors. Nihon-retto no kankyo-shi (Environmental records of Japan archipelago), Vol. 6. Tokyo: Bun-ichi Shuppan. (2011) p. 85–103 [3].

## Value of the Data

• This data is useful to reconstruct regional variation of palaeodiet in premodern Japan.
• This data can benefit archaeologists, anthropologists, and historians interested in palaeodietary reconstruction.
• This data can be used for international comparison of premodern diets among different regions. Also, more efficient sampling and stable isotope analyses are possible to understand the overview of the dietary habits of premodern Japan archipelago by considering the regions with few stable isotopic data reported.
• Stable isotope ratios previously reported either in excavation report, book chapter, or museum bulletin were compiled to this dataset. Most of them were written in Japanese, without a digital object identifier, and reported only in Figs. without raw data tables. This compilation of the data enables easier access.

## 1 Data Description

**Fig. 1:**
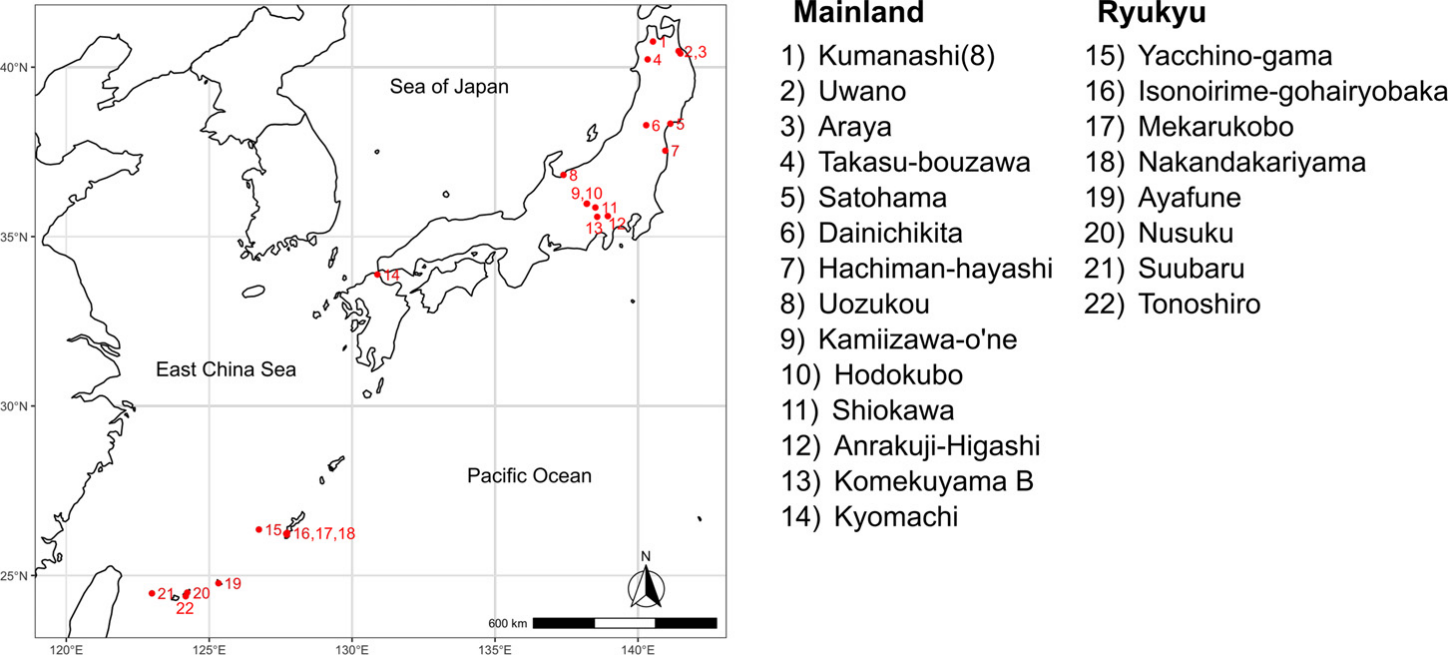
Map of the location of the included archaeological sites. The map was drawn with the R package “rnaturalearth” [8].

This dataset consists of carbon and nitrogen stable isotope ratios (δ^13^C and δ^15^N values, respectively) of bulk collagen extracted from premodern human skeletons from mainland Japan and the Ryukyu Islands. There are a number of archaeological sites that yielded premodern human skeletons in Japan. Among such premodern human skeletal collections from Japan, accessible ones were selected and stable isotopic data were collected by the second author (Fig. 1). The diet of consumers, such as humans, can be estimated and compared through the carbon and nitrogen stable isotope analysis of their tissues, such as bone collagen.

**Fig. 2:**
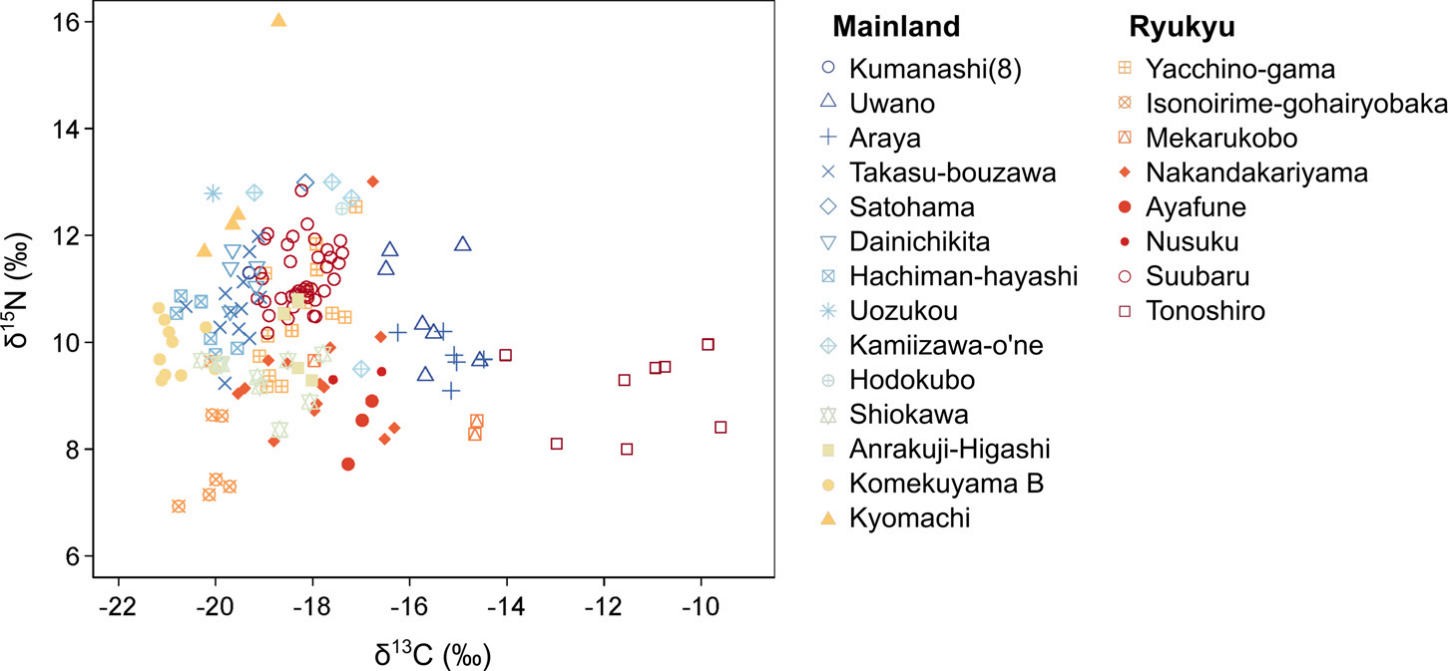
Carbon and nitrogen stable isotope ratios of collagen with acceptable C/N ratio extracted from 166 skeletal individuals in premodern Japan.

Archaeological contexts of the target sites are summarized in Table 1. Stable isotopic data of 229 human skeletal individuals from 22 archaeological sites were compiled in this dataset (Fig. 2). Archaeological ages of the sites or individual graves were mostly determined from the typology of excavated remains, such as coins, ceramics, and grave stones. Skeletal individuals from mainland Japan were mostly dated to the Edo period (AD 1603−1867), but some individuals possibly came from the late 19th century. Skeletal individuals from the Ryukyu Islands were mostly dated to the Ryukyu Kingdom period (AD 1429−1879). These periods are regarded as premodern periods in mainland Japan and the Ryukyu Islands, respectively.

**Table 1:** Details of the archaeological sites included in this dataset. If the isotopic data (raw data or figures) of human skeletons have been reported previously, references were shown. Note that these references are written in Japanese.

Site	Japanese site name	Period	Prefecture	Latitude	Longitude	Altitude	Type of coordinates	N. all individuals	N. acceptable individuals	References
Kumanashi (8)	隈無 (8)	After Late 17th century	Aomori	40.757	140.525	20.5	Exact	7	1	[3,9]
Uwano	上野	Late 17th−18th century	Aomori	40.475	141.421	30	Exact	12	7	[3]
Araya	荒谷	Premodern	Aomori	40.404	141.483	200	Approximate	25	6	[3]
Takasu-bouzawa	鷹巣坊沢	Premodern	Akita	40.230	140.338	94	Approximate	12	12	[3]
Satohama	里浜	Premodern	Miyagi	38.333	141.133	0	Approximate	1	1	[3]
Dainichikita	大日北	Late 17th−Late 18th century	Miyagi	38.288	140.288	101	Exact	5	5	[3]
Hachiman-hayashi	八幡林	Premodern	Fukushima	37.535	140.957	95	Approximate	7	6	[3]
Uozukou	魚津港	195 ^14^C BP	Toyama	36.821	137.394	2	Approximate	1	1	[3,10]
Kamiizawa-o'ne	上居沢尾根	Premodern	Nagano	35.974	138.216	955	Exact	4	4	[3,11]
Hodokubo	程久保	Premodern	Nagano	35.968	138.205	960	Exact	1	1	[3,11]
Shiokawa	塩川	18th−19th century	Yamanashi	35.859	138.509	843	Exact	10	10	[3]
Anrakuji-Higashi	安楽寺東	Late 18th−Early 20th century	Yamanashi	35.605	138.944	366	Exact	9	5	[3,12]
Komekuyama B	米倉山B	17th−18th century	Yamanashi	35.584	138.573	380	Approximate	10	10	[3]
Kyomachi	京町	Late 17th−Early 19th century	Fukuoka	33.881	130.885	10	Exact	10	5	[3]
Yacchino-gama	ヤッチのガマ	Middle 18th century	Okinawa	26.355	126.739	8	Exact	26	16	[3,6,13]
Isonoirime-gohairyobaka	伊祖の入め御拝領墓	AD 1665−1927	Okinawa	26.256	127.727	55	Exact	20	7	[3,6]
Mekarukobo	銘苅古墓群	17th−20th century	Okinawa	26.234	127.696	28	Exact	3	3	[3,6]
Nakandakariyama	ナカンダカリヤマ	16th−17th century	Okinawa	26.200	127.717	673	Approximate	14	14	[3,6]
Ayafune	綾船	Premodern	Okinawa	24.729	125.364	37	Approximate	3	3	[3,6]
Nusuku	野底	18th−19th century	Okinawa	24.494	124.232	20	Approximate	2	2	[3,6]
Suubaru	潮原	Premodern−Modern	Okinawa	24.471	122.996	24	Exact	39	39	[3,6]
Tonoshiro	登野城	15th−18th century	Okinawa	24.386	124.178	3.5	Approximate	8	8	[3,6]

**Fig. 3:**
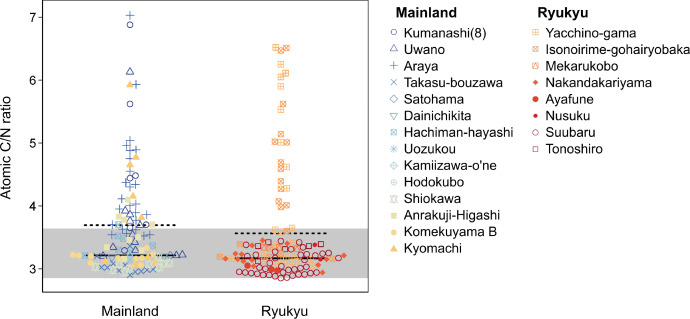
Atomic C/N ratio of collagen extracted from 229 skeletal individuals in premodern Japan. The acceptable range (2.9−3.6) of the C/N ratio is shown with a gray band. Mean C/N ratios of acceptable and all individuals from mainland Japan (3.21 ± 0.19 and 3.69 ± 0.83) and Ryukyu Islands (3.17 ± 0.18 and 3.56 ± 0.90) are shown with horizontal solid and dotted lines, respectively.

Individual data includes δ^13^C and δ^15^N values and atomic carbon-to-nitrogen ratio (C/N ratio), at least. Quality indicators of collagen preservation (i.e., atomic concentration of carbon and nitrogen and collagen yield) and physical anthropology measures (e.g., age, sex, and skeletal element sampled) were shown if such information was available. Samples with atomic C/N ratios within the acceptable range (i.e., 2.9−3.6) are regarded to provide reliable stable isotope ratios [4]. Samples with unreliable C/N ratios are also included in this dataset (Fig. 3), but should be omitted for further analyses. Eleven samples with unknown C/N ratios were regarded as acceptable and shown in Fig. 2.

All collected data represents stable isotopic data from single human (*H. sapiens*) individuals without duplication. Most data was derived from adults (i.e., skeletons aged older than puberty). Because the δ^13^C and δ^15^N values of breastfed infants systematically differ compared with those of adults due to the trophic level enrichment between mother and infant [5], data from infants should be analyzed separately from those from adults. This dataset was presented in previous literature written in Japanese [3,6] but only shown in figures without raw data.

## 2 Experimental Design, Materials and Methods

Approximately 10 individuals were randomly selected from each archaeological site to investigate regional variation of diet rather than intra-population variation. Adult individuals were prioritized. Most of such skeletal individuals were identified, sexed, and aged by physical anthropologists. Skeletal element that has relatively fewer morphological information, such as ribs, were selectively used for the stable isotope analysis.

Collagen protein was extracted from the sampled skeletal elements following the “powder” method described in a previous research [1]. At first, 0.2−0.5 g of bone pieces, or dentin pieces in a few cases, were cut from the skeletal element. In order to avoid breastfeeding effect [5], root tip of dentin, which represent older ages during the tooth development, was sampled in case of dentin samples. Surface of the bone/dentin pieces was mechanically cleaned by sandblasting of baked aluminum oxide powder. The cleaned bone/dentin pieces were soaked in Milli-Q water under ultrasonication at 4°C for 10 min and then 0.2 M NaOH at 4°C overnight to remove exogenous organic matter, such as humic acids. The samples were rinsed with Milli-Q water, and then freeze-dried and crushed into coarse powders. The crushed samples were sealed in cellulose tubes and treated with 1.0 M HCl at 4°C overnight for demineralization. The HCl solution was replaced with Milli-Q water by dialysis. The demineralized remaining portion was recovered from cellulose tubes and concentrated by centrifuging. The samples were then gelatinized in Milli-Q water at 90 °C for 12 h, filtered using a glass fiber filter (Wattmann GF/F), and freeze-dried. Although typical collagen extraction protocols apply NaOH-treatment after the demineralization step, collagen samples of this dataset were treated with NaOH before the demineralization step. However, no significant difference in δ^13^C and δ^15^N values was observed between collagen samples extracted with these different protocols [7]. Therefore, this data is comparable with other stable isotopic datasets that adopted NaOH-treatment after the demineralization step.

Carbon and nitrogen stable isotope ratios were measured using an EA-IRMS (Thermo Flash 2000 elemental analyzer, Finnigan ConFlo III interface, and Thermo Delta V mass spectrometer) at the University Museum, University of Tokyo, Japan or an EA-IRMS (Calro Erba NA1500 elemental analyzer, Finnigan MAT ConFlo II interface, and Finnigan MAT 252 mass spectrometer) at National Institute for Environmental Studies, Ibaragi, Japan. The δ^13^C and δ^15^N values were calibrated against the laboratory working standards (L-alanine 1: δ^13^C = −18.5 ± 0.2‰, δ^15^N = -1.02 ± 0.2‰; L-alanine 2: δ^13^C = −19.6 ± 0.2‰, δ^15^N = 8.7 ± 0.2‰; L-alanine 3: δ^13^C = −19.6 ± 0.2‰; δ^15^N = 20.0 ± 0.2‰; L-histidine: δ^13^C = −7.6 ± 0.2‰, δ^15^N = 11.4 ± 0.2‰) provided by SI Science Co. (Saitama, Japan), whose values were determined by the NBS 19 and the International Atomic Energy Agency (IAEA) Sucrose ANU (calibrated against Vienna Pee Dee Belemnite) and IAEA N1 and IAEA N2 (calibrated against AIR) international standards, respectively. Based on repeated measurements of the calibration standard, precision was determined to be less than ±0.1‰ and ±0.2‰ standard deviation (SD) for δ^13^C and δ^15^N values, respectively.

## Ethics Statement

Use of the archaeological human skeleton were approved by appropriate organizations or persons, such as the collection managers, governmental board of education, and descendant communities.

## CRediT Author Statement

**Takumi Tsutaya:** Conceptualization, Methodology, Software, Validation, Investigation, Data Curation, Writing, Visualization; **Minoru Yoneda**: Conceptualization, Methodology, Validation, Resources, Investigation, Data Curation, Project administration, Funding acquisition.

## Declaration of Competing Interest

The authors declare no competing interest.
